# Cuproptosis-related LncRNAs signature as biomarker of prognosis and immune infiltration in pancreatic cancer

**DOI:** 10.3389/fgene.2023.1049454

**Published:** 2023-01-13

**Authors:** Hui Chen, Yang Yu, Lei Zhou, Junliang Chen, Zeyu Li, Xiaodong Tan

**Affiliations:** Department of General Surgery, Pancreatic, and Thyroid Ward, Shengjing Hospital of China Medical University, Shenyang, Liaoning, China

**Keywords:** pancreatic cancer, cuproptosis, lncRNA signature, mutation landscape, tumor microenvironment

## Abstract

**Background:** Pancreatic cancer (PC) is a malignant gastrointestinal tumor with a terrible prognosis. Cuproptosis is a recently discovered form of cell death. This study is intended to explore the relationship between cuproptosis-related lncRNAs (CRLncs) signature with the prognosis and the tumor microenvironment (TME) of PC.

**Methods:** Transcript sequencing data of PC samples with clinical information were obtained from the Cancer Genome Atlas (TCGA). Univariate Cox regression analysis and LASSO regression analysis were employed to construct the prognostic signature based on CRLncs associated with PC survival. A nomogram was created according to this signature, and the signaling pathway enrichment was analyzed. Subsequently, we explored the link between this prognostic signature with the mutational landscape and TME. Eventually, drug sensitivity was predicted based on this signature.

**Results:** Forty-six of 159 CRLncs were most significantly relevant to the prognosis of PC, and a 6-lncRNA prognostic signature was established. The expression level of signature lncRNAs were detected in PC cell lines. The AUC value of the ROC curve for this risk score predicting 5-year survival in PC was .944, which was an independent prognostic factor for PC. The risk score was tightly related to the mutational pattern of PC, especially the driver genes of PC. Single-sample gene set enrichment analysis (ssGSEA) demonstrated a significant correlation between signature with the TME of PC. Ultimately, compounds were measured for therapy in high-risk and low-risk PC patients, respectively.

**Conclusion:** A prognostic signature of CRLncs for PC was established in the current study, which may serve as a promising marker for the outcomes of PC patients and has important forecasting roles for gene mutations, immune cell infiltration, and drug sensitivity in PC.

## 1 Introduction

The number of newly diagnosed pancreatic cancer (PC) in the United States broke 60,000 in 2021, with the incidence increasing by over 1% annually. After numerous years of efforts worldwide, the 5-year survival rate for PC has only just surpassed 10% (Rahib et al., 2014; Siegel et al., 2021; Siegel et al., 2022). The risk elements for PC are not well-defined and the main ones generally accepted are family history, tobacco, alcohol abuse, obesity, and type II diabetes (Mizrahi et al., 2020; Huang et al., 2021). Lacking effective early screening measures, localized PC patients often are asymptomatic or have minimal symptoms, and approximately half of newly diagnosed PC cases are advanced diseases at diagnosis, with an average survival time of less than 1 year (Mizrahi et al., 2020). The high heterogeneity of PC contributes to the challenge of predicting its prognosis, and satisfactory results are not achievable by traditional TNM staging alone. Given the current dilemma in the prognostic prediction of PC, new strategies for screening high-risk patients to detect PC at an early stage are urgently necessary to provide practical clinical benefit (Mizrahi et al., 2020). Hence, a prognostic model based on molecular profiling to screen for high-risk patients is appreciated.

Tsvetkov et al. found that copper triggered the aggregation of mitochondrial lipid acylated proteins to induce regulated cell death (RCD) and named this unique form of death “cuproptosis” (Tang et al., 2022; Tsvetkov et al., 2022). Studies have been proposed before this to suggest a strong relationship between copper with cancer. Yu et al. suggested that blocking SLC31A1-dependent copper uptake increased PC cell autophagy to resist cell death (Yu et al., 2019). Inhibition of copper transport induces apoptosis and inhibits tumor angiogenesis in triple-negative breast cancer cells (Karginova et al., 2019). Of course, the link between copper with tumor is not only in the cuproptosis but also as a possible therapeutic target for treatment (Safi et al., 2014). Disulfiram (DSF) acts as a copper carrier to induce copper-dependent oxidative stress and mediates antitumor efficacy in inflammatory breast cancer (Allensworth et al., 2015). Thus, this form of death brings promise for the therapy of PC.

Long non-coding RNAs (LncRNAs) are described as RNAs consisting of over 200 nucleotides without the ability to encode proteins (Gibb et al., 2011; Serghiou et al., 2016). LncRNAs exert a central role in PC pathogenesis as regulators of cancer pathways, regulating cellular processes including but not limited to cell cycle, apoptosis, and epithelial-mesenchymal transition to influence biological behaviors such as tumor growth, migration, and invasion (Kornienko et al., 2013; Sharma et al., 2021). The critical role of LncRNAs in cancer is what makes them proposed as important biomarkers of cancer outcome and are emerging as promising candidates for biomarker development in various cancers, including PC (Esteller, 2011; Sharma et al., 2021; Toden et al., 2021). The potential value of prognostic signature regarding cuproptosis-associated lncRNAs as a prognostic predictor for PC is currently uncertain.

The current study utilized CRLncs to create a signature for PC as well as a nomogram to predict the prognosis of PC patients. The intimate association of signature with mutational pattern in PC samples and immune infiltration landscape in TME was revealed. In this study, the high expression of signature lncRNAs in PC cell lines was investigated by PCR experiment. This study identified a prognostic signature of CRLncs as a potentially promising marker that could be applied for forecasting the long-term survival time and guiding the therapeutic regimen for PC patients.

## 2 Materials and methods

### 2.1 Data source

Transcript sequencing data and clinical details of PC patients were available in the latest version of TCGA (July 2022, https://portal.gdc.cancer.gov/). The raw copy number variation (CNV) data of PC was extracted from UCSC Xena (http://xena.ucsc.edu/). Nineteen cuproptosis-related genes (CRGs) (Supplementary Table S1) were searched from works of literature (Yu et al., 2019; Gao et al., 2021; Tang et al., 2022).

### 2.2 To identify CRLncs

The “igraph” package was utilized to draw the correlations within CRGs. The cuproptosis-related lncRNAs were identified *via* Pearson correlation analysis with a correlation coefficient = .35 and *p* = .05 considered relevant.

### 2.3 CRLncs prognostic signature

First, the prognostic value of CRLncs was assessed by univariate Cox analysis, whereby a threshold of cox = .05 and *p* < .05 was accepted. In the current study, The least absolute shrinkage and selection operator (LASSO) Cox regression algorithm was employed to remove overfitting between CRLncs and construct CRLncs signature, which was run through the “glmnet” package (Engebretsen and Bohlin, 2019). Risk score formula:

$$Risk\;score=\overset{n}{\underset{i}{\sum }}\left(CoefCRLncs\times ExpCRLncs\right)$$

The TCGA samples were randomly divided into train cohort and test cohort initially. The risk of PC samples was scored and patients were classified as high-risk and low-risk groups with a cut-off of median risk. Kaplan-Meier (K-M) survival curves were plotted to assess the progression-free survival (PFS) and overall survival (OS) of PC patients. Time-dependent receiver operating characteristic (ROC) curves were drawn *via* the “timeROC” package to determine the performance of this signature. Principal Components Analysis (PCA) was performed for each risk subtype by the “scatterplot3d” package.

### 2.4 To construct a nomogram

Univariate combined with multivariate Cox regression analyses to determine whether this risk score is a prognostic factor independently of other clinical features. A nomogram was constructed with the “rms” package to predict the survival rate of individual patients, and calibration curves were employed to measure the performance of the nomogram model. The concordance index (C-index) curve was plotted to detect the stability of the risk score and nomogram prediction power. The decision curve analysis (DCA) was carried out to determine the contribution of risk score and nomogram in clinical decision-making.

### 2.5 Functional enrichment analysis

Initially, differences in active signaling pathways among different risk PC samples were explored by Gene Set Enrichment Analysis (GSEA). To understand the biological functions of risk-related differential genes and the potential signaling enrichment pathways, the gene ontology (GO) enrichment analysis and Kyoto Encyclopedia of Genes and Genomes (KEGG) pathway enrichment analysis were conducted with the package of “clusterProfiler”, and presented as bar graphs, bubble plots, and functional clustering circles, respectively.

### 2.6 Mutation landscape

Firstly, the “maftools” R package was employed to describe the mutational patterns of CRGs. The “maftools” and “Rcircos” packages were employed for CNV analysis. All mutation frequencies and oncoplot waterfall maps of PC patients were drawn by the “maftools” package. The association between risk score and the frequency of mutations in the classical driver genes of PC (KRAS, TP53, SMAD4, and CDKN2A) was further explored (Kanda et al., 2012). The association between TMB and patient OS was analyzed in a tumor mutation load analysis, and the “maftools” package was finally utilized to compare the mutation frequency between different risk groups.

### 2.7 Immune infiltration analysis

First, the CIBERSORT algorithm was used to reveal the immune infiltration pattern of PC samples in TCGA, and the “corrplot” package was utilized to explore the correlation among various immune cells. Alternatively, the ssGSEA was employed to compare differences in the level of immune cell infiltration and immune function in different risk PC samples. Eventually, the expression levels of HLA family genes and immune checkpoint genes were compared between patients with different risks.

### 2.8 Compound sensitivity analysis

To investigate the potential of this risk score for PC therapy, we explored the drug therapy data in the Cancer Therapeutics Response Portal (CTRP) database with the “oncoPredict” package (Maeser et al., 2021). This study focused on the differences in sensitivity of PC patients with different risk profiles to compounds currently in clinical use. Through literature review, we collected 24 compounds that are at least in the clinical trial stage for PC clinical treatment (Sally et al., 2022).

### 2.9 Cell culture

The pancreatic cancer cell lines of AsPC-1, Capan-2, CFPAC-1, MIA PaCa-2, and SW1990 were used to detect the expression levels of lncRNAs, and HPDE cell lines were employed as normal controls. AsPC-1, Capan-2, and CFPAC-1 were cultured with 1,640 complete medium, while MIA PaCa-2 and SW1990 were cultured with DMEM complete medium.

### 2.10 Quantitative real-time PCR

Details of the full procedure of the PCR operation were carried out in accordance with previous study (30333874). PCR primers for FAM83A-AS1, PAN3-AS1, and SENCR were purchased from SangonBiotech (Sangon, Shanghai, China). The primer sequences were as follows: FAM83A-AS1: 5’ -AAG AGC ATG AAA GAC TGA GGA AGC G-3’ (forward), 5’ -TCC AGG AGG TCG GTG CCA TTG-3’ (reverse); PAN3-AS1: 5’ -CTT CCT CTC CCC GTT TCC TTT CTT C-3’ (forward), 5’ -CAA GAG GTT AGC GTA ATC GGT CCA G-3’ (reverse); SENCR: 5’ -GCT TTC AGG AGA ATG CGG AGA GAC-3’ (forward), 5’ -TTC TGG CTG AAT GAG GAG CAA TGT G-3’ (reverse); β-actin: 5’ -CCT GGC ACC CAG CAC AAT-3’ (forward), 5’ -GGG CCG GAC TCG TCA TAC-3’ (reverse). The expression of FAM83A-AS1, PAN3-AS1, and SENCR was standardized by the internal control β-actin. In addition, fold changes in FAM83A-AS1, PAN3-AS1, and SENCR were calculated by 2^−ΔΔCT^.

## 3 Statistical analysis

All analysis processes were carried out with the R software (https://www.r-project.org/, version 4.2.1) plus its application packages. The Student’s t-test was employed to compare the statistical difference between two groups. The log-rank test was performed for the Kaplan-Meier survival analysis. Drug sensitivity differences were compared with the Wilcoxon test. *p*-value <.05 is considered statistically significant if not otherwise stated. *p < .05, **p < .01; ***p < .001; ns, not significant.

## 4 Results

### 4.1 Genetic variation of CRGs in PC

**FIGURE 1:**
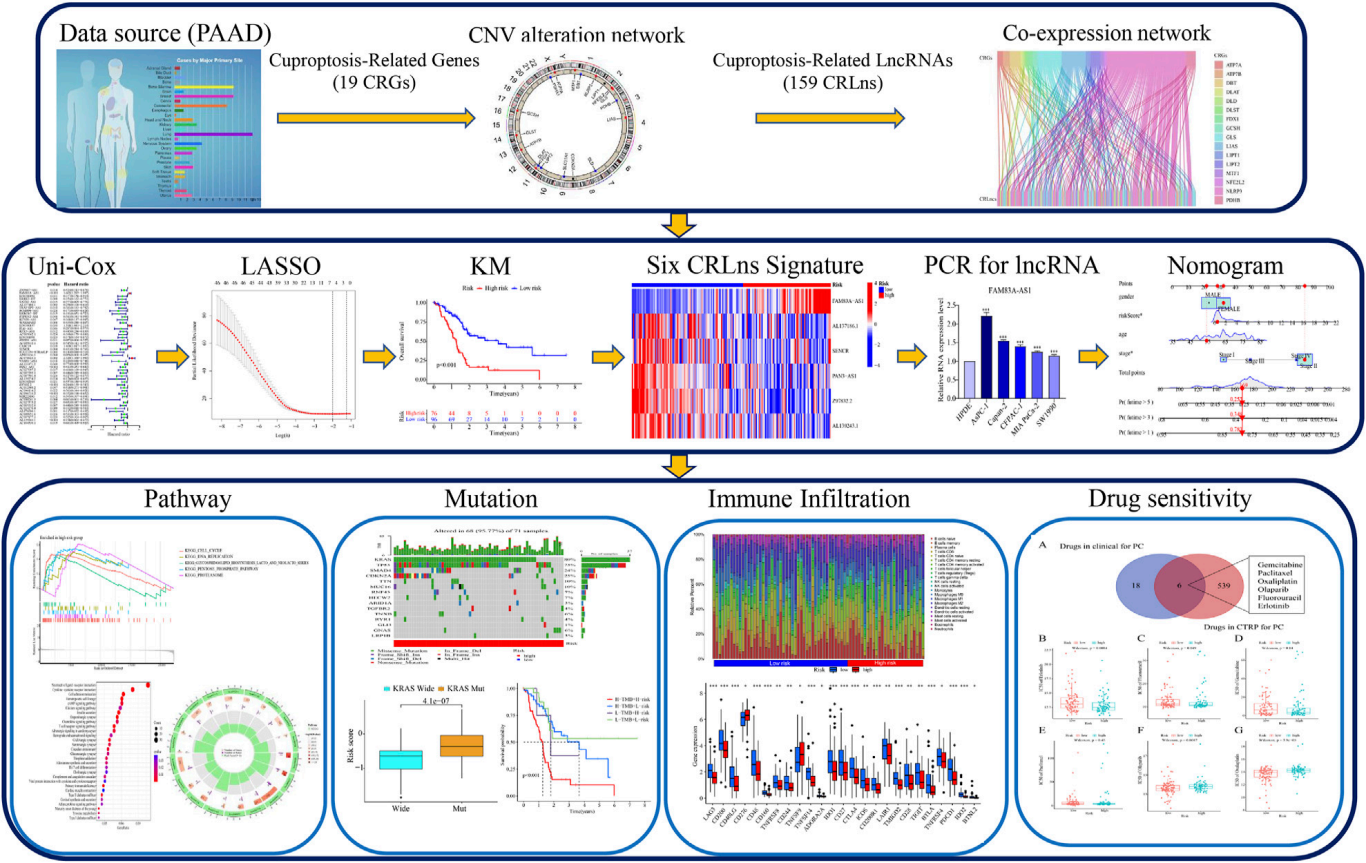
The flowchart for analyzing cuproptosis-related lncRNAs prognostic signature.

**FIGURE 2:**
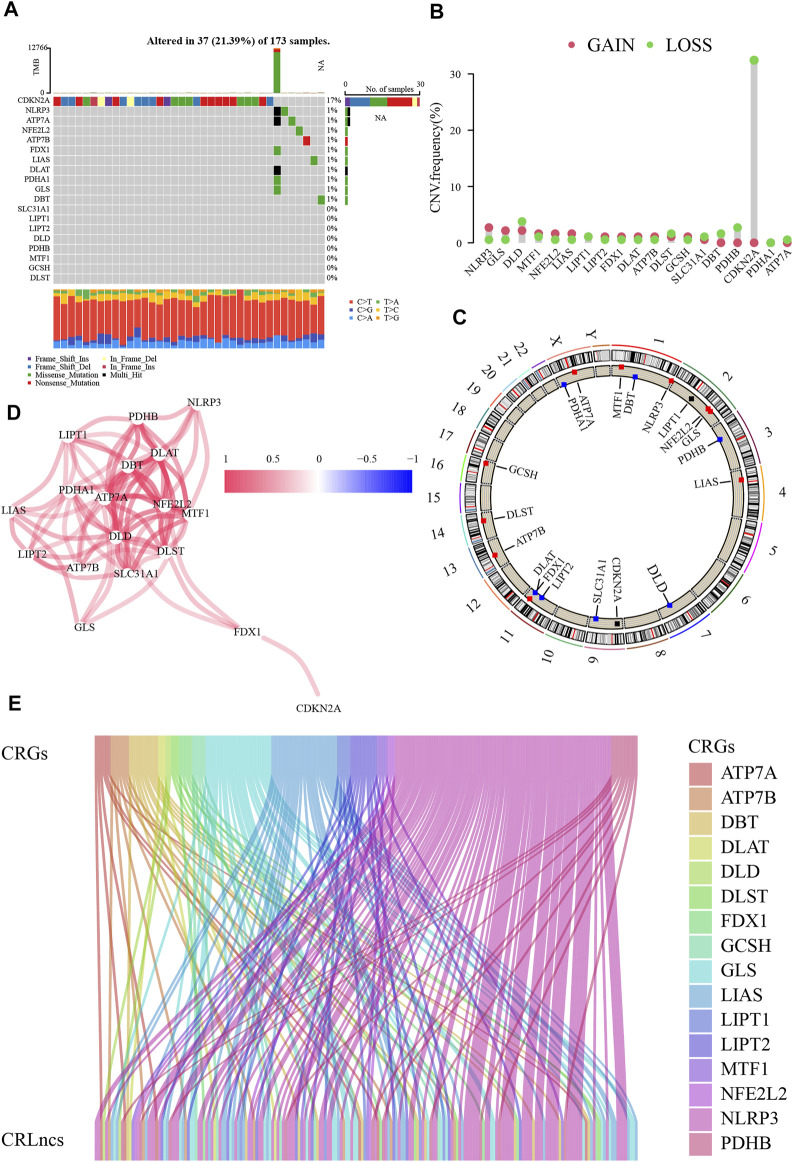
Genetic variations of cuproptosis-related genes (CRGs). **(A)** The mutation waterfall plot of the CRGs in PC. **(B)** Copy number variation (CNV) frequencies of CRGs. **(C)** The sites of CRGs with CNV on chromosomes. **(D)** The interconnections among CRGs. **(E)** Identification of cuproptosis-related lncRNAs (CRLncs).

This study progressed according to the flow chart (Figure 1). The TCGA dataset of 179 PC was downloaded, accompanied by four normal pancreatic tissue samples. The mutation waterfall plot of the nineteen CRGs in PC samples indicated that the frequency of CDKN2A mutation was the highest (17%), NLRP3, ATP7A, NFE2L2, ATP7B, FDX1, LIAS, DLAT, PDHA1, GLS, and DBT were mutated at 1%, and the remaining were unmutated (Figure 2A). The genetic instability of CRGs in PC samples is commonly visible (Figures 2B, C). The interconnections among these CRGs are very obvious (Figure 2D).

### 4.2 Establishing and validating a CRLncs signature

**FIGURE 3:**
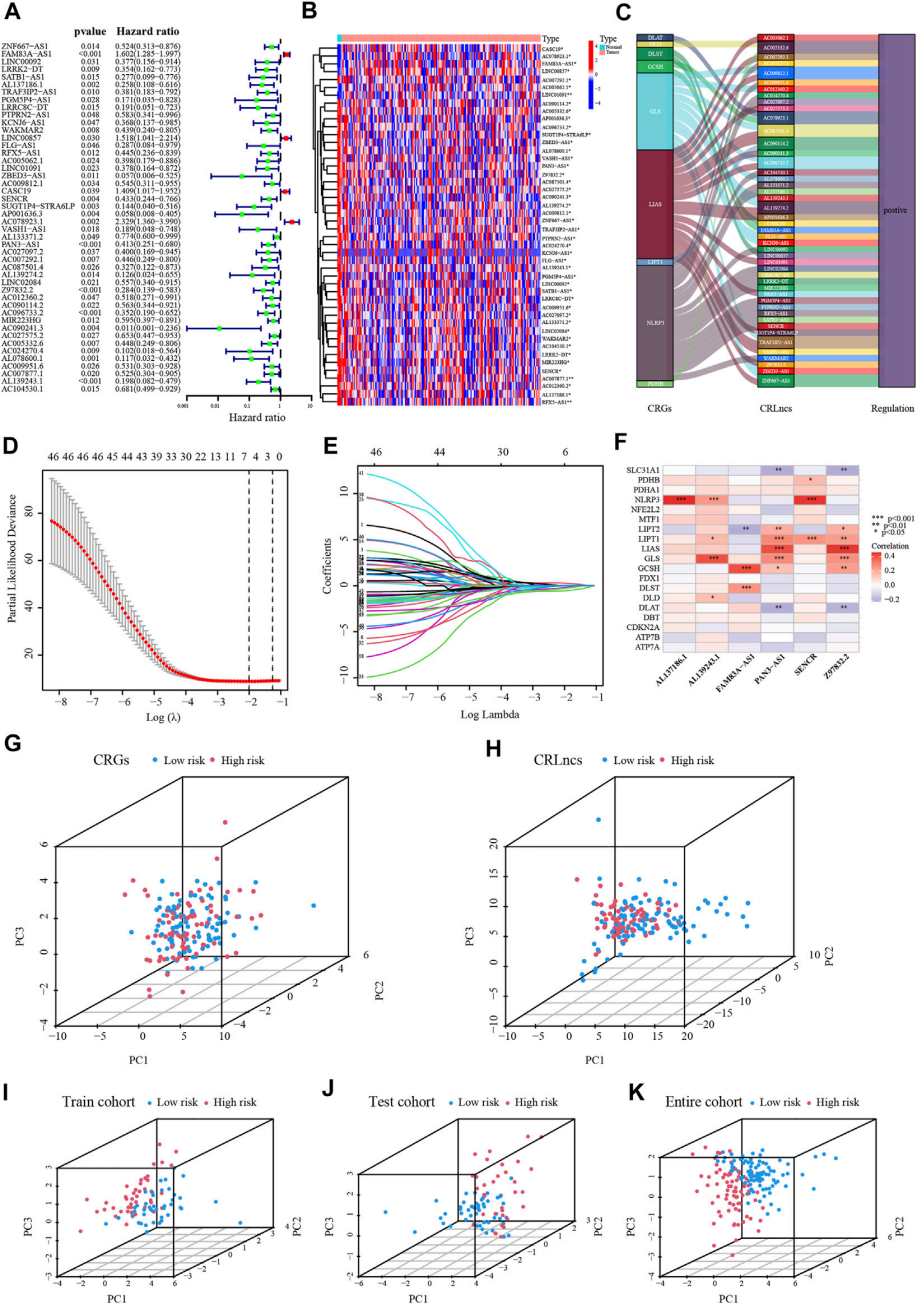
Establishment of the CRLncs signature for PC. **(A)** Recognition of the prognostic CRLncs in PC *via* univariate Cox regression analysis. **(B)** Heatmap of the expression of prognostic CRLncs. **(C)** The ggalluvial diagram of relationship between CRGs and prognostic CRLncs. **(D,E)** The LASSO Cox regression analysis was performed depending on the optimal λ value. **(F)** The relationship between CRGs with signature CRLncs. **(G–K)** The PCA of PC patients in the TCGA-train, test, and entire cohort. *p < .05, **p < .01; ***p < .001; ns, not significant.

In the TCGA dataset, altogether 159 CRLncs were identified (Figure 2E). Initially, forty-six of the above CRLncs were identified as prognostically associated with PC using univariate Cox regression analysis, of which four were risk factors and the rest were protective factors (Figure 3A). The heatmap showed that all 46 CRLncs mentioned above were differentially expressed in PC tissues (Figure 3B). A strong positive regulatory relationship was observed between these CRLncs and CRGs, with most CRLncs being closely related to GLS, LIAS, and NLRP3 (Figure 3C). Subsequently, LASSO Cox regression analysis was performed to obtain a CRLncs signature with an optimal λ value (Figures 3D, E). The signature is the sum of the product of the coefficient of the six CRLncs with their expression, as follows:

$$Risk\;score=\left[0.2738*expression\;of\;FAM83A-AS1\right]+\left[-0.2123*\;expression\;of\;AL137186.1\right]+\left[-0.0024*expression\;of\;SENCR\right]+\left[-0.0193*expression\;of\;PAN3-AS1\right]+\left[-0.5518*expression\;of\;Z97832.2\right]+\left[-0.2967*expression\;of\;AL139243.1\right]$$

**FIGURE 4:**
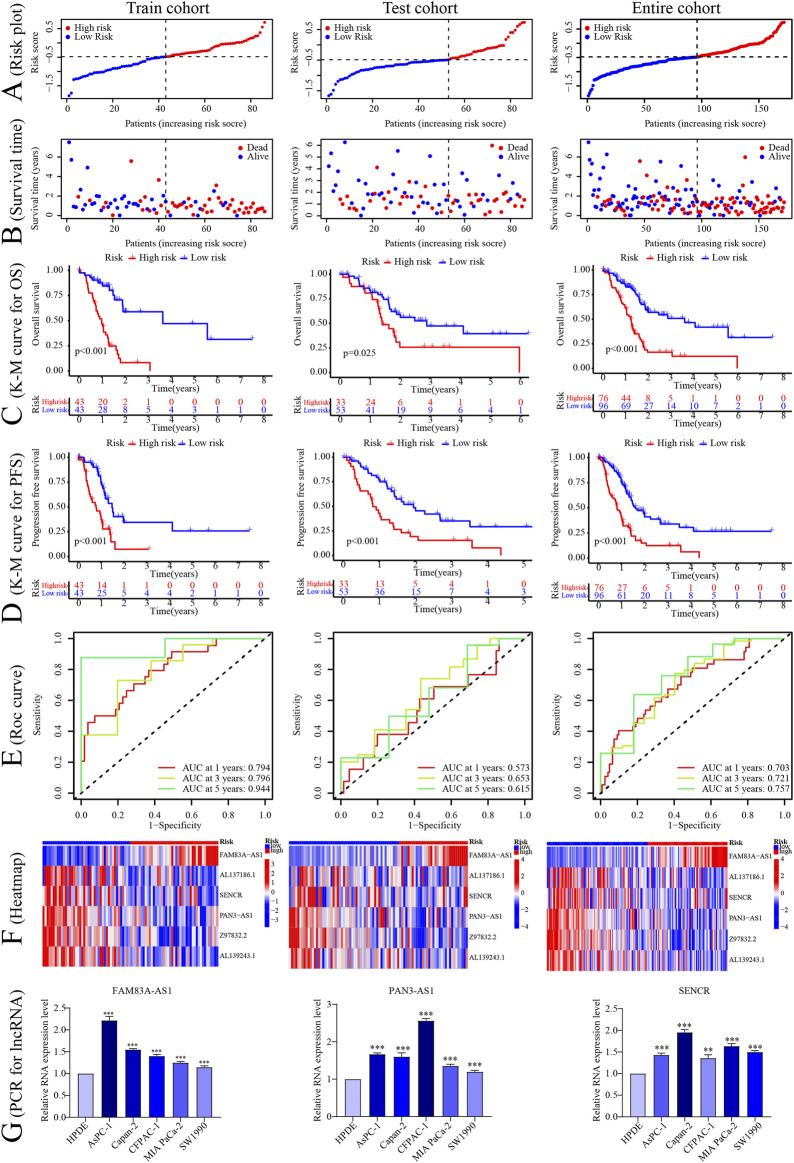
Construction of the CRLncs prognostic signature. **(A–F)** The risk score, survival status, Kaplan-Meier plot survival cure of OS and PFS, ROC curve predicted the 1/3/5-year OS, and heatmap of 6 signature CRLncs in different risk PC patients in the TCGA-train, test, and entire cohort. **(G)** The PCR of signature lncRNAs expression levels. *p < .05, **p < .01; ***p < .001; ns, not significant.

According to this model, the risk score was calculated for individual patients in the training cohort, the validation cohort, and the entire TCGA cohort. PC patients were divided into high- and low-risk groups based on the median risk score. The correlation between the six signature CRLncs with CRGs was next assessed, with distinct positive relationships between AL137186.1, AL139243.1, and SENCR with NLRP3. There was a significant positive connection between FAM83A-AS1 with GCSH and PAN3-AS1 with Z97832.2 (Figure 3F). PCA analysis demonstrated that the high-risk samples in the three cohorts were independent of the low-risk samples (Figures 3G–K). The risk score and survival status map were plotted (Figures 4A, B). As the K-M curves suggested, low-risk patients shared significantly superior OS and PFS to high-risk patients (Figures 4C, D). The area under the ROC curve (AUC) values for 1/3/5 years were 0.794/0.796/0.944 in the training cohort, 0.573/0.653/0.615 in the validation cohort, and 0.703/0.721/0.757 in the whole cohort, respectively (Figure 4E). The expression of six signature CRLncs was substantially different between the high- and low-risk groups, with FAM83A-AS1 being more highly expressed in the high-risk group and the remaining being expressed at higher levels in the opposite risk group (Figure 4F). High expression of FAM83A-AS1, PAN3-AS1, and SENCR in PC cell lines was demonstrated by PCR (Figure 4G).

### 4.3 Building a nomogram

**FIGURE 5:**
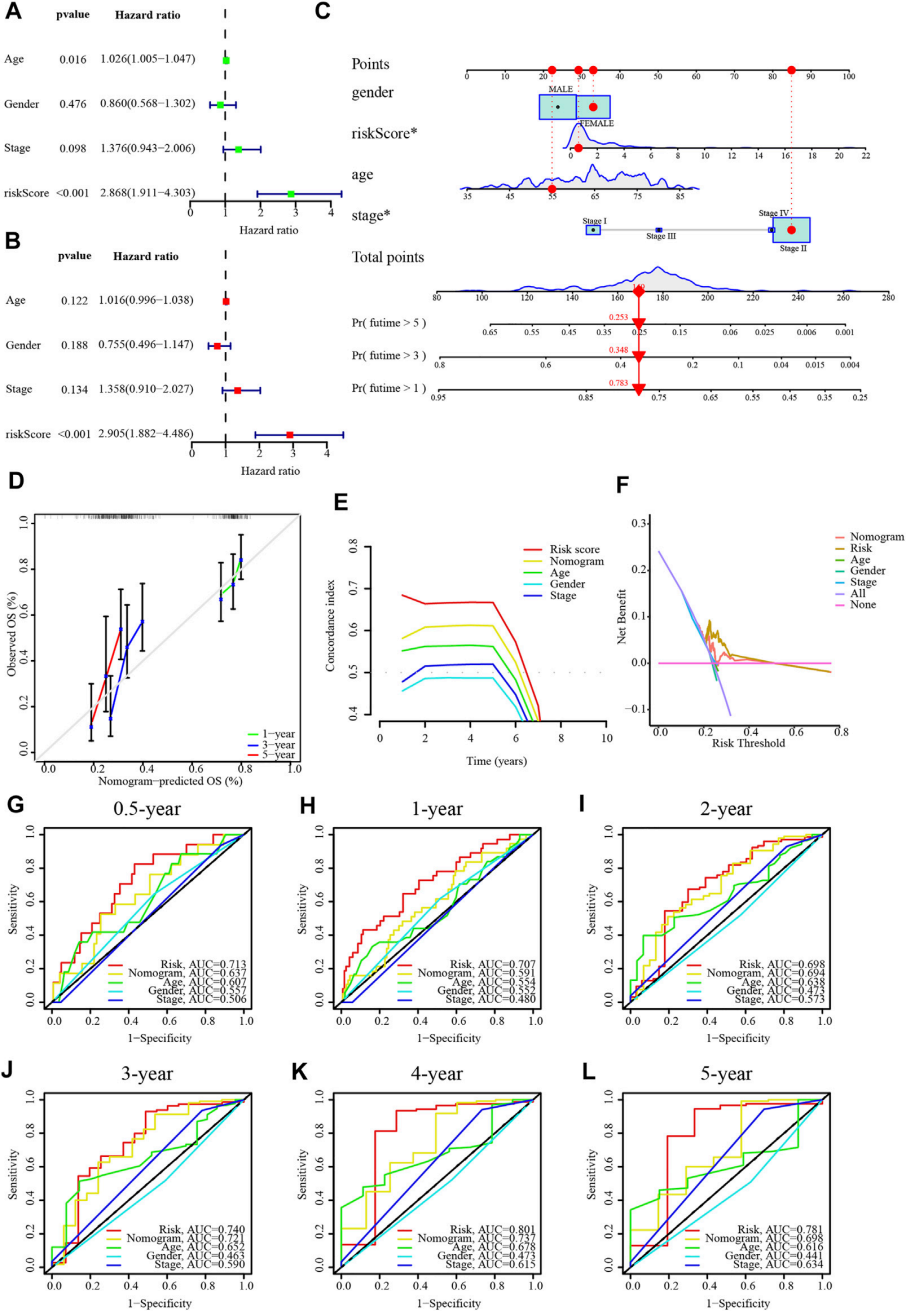
A nomogram for PC patients in TCGA dataset. **(A,B)** Independent prognostic value of risk score by univariate and multivariate analysis. **(C)** A nomogram of the risk score for predicting the 1-, 3-, and 5-year OS of PC patients. **(D)** Calibration curves of this nomogram for the prediction of 1/3/5-year OS of PC patients. **(E)** The concordance index curve of risk score and nomogram. **(F)** The DCA curve of risk score and nomogram. **(G–L)** The AUC values for risk score and nomogram predict 0.5/1/2/3/4/5-year OS. *p < .05, **p < .01; ***p < .001; ns, not significant.

**FIGURE 6:**
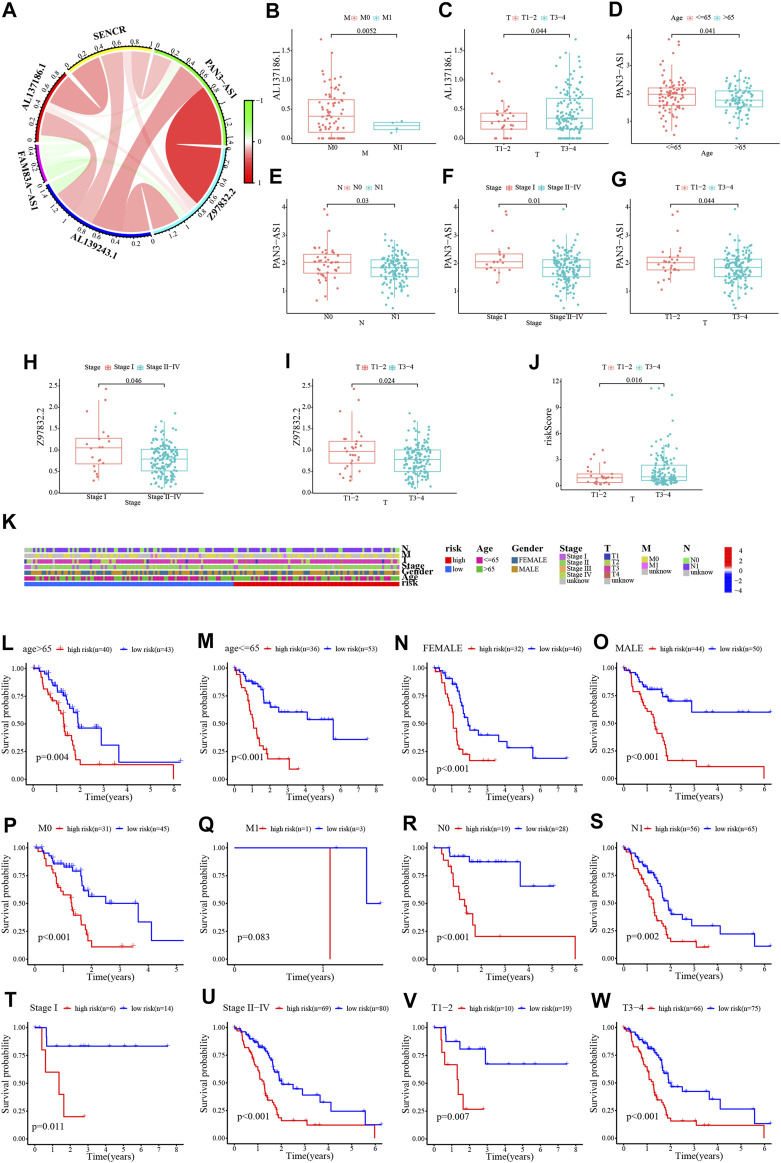
Relationship between risk score and clinical characteristics. **(A)** Chord diagram of the relationship between the six signature CRLncs. **(B–J)** The relationship between these signature CRLncs and risk score with clinical features. **(K)** The clinical characteristics heatmap. **(L–W)** The survival of PC patients in different risk group across clinical characteristic subgroups.

The results of univariate and multivariate Cox regression analyses suggested that this risk score was an independent prognostic factor for PC (Figures 5A, B). Alternatively, a nomogram was generated to score patients and predict their OS in 1, 3, and 5 years (Figure 5C), and the calibration curve showed that the nomogram performed well (Figure 5D). The concordance index curve demonstrated that risk score and nomogram outperformed age, gender, and stage (Figure 5E). The DCA curve demonstrated the contribution of risk and nomogram in clinical decision-making (Figure 5F). Consistently, the AUC values for risk score predicting 0.5/1/2/3/4/5-year overall survival were .713, .707, .698, .740, .801, and .781, respectively, and the AUC values for nomogram predicting .5/1/2/3/4/5-year OS were .637, .591, .694, .721, .737, and .698, which were better than other clinical characteristics (Figures 5G–L). Further, the interrelationship between the signature CRLncs was assessed and a remarkable positive correlation was found between PAN3-AS1 with Z97832.2 (Figure 6A). The relationship between these signature CRLncs and risk score with clinical features is intimate (Table 1). AL137186.1 was highly expressed in patients with M0 or T3-4 (Figures 6B, C). PAN3-AS1 was more abundant in younger or N0 patients (Figures 6D, E). PAN3-AS1, Z97832.2, and risk score were expressed at higher levels in patients with high stage and T-staging (Figures 6F–J). The heat map illustrates the distribution of risk score with clinical characteristics (Figure 6K). The outcomes of PC patients in the low-risk group were better than their counterparts, across all clinical characteristic subgroups (Figures 6L–W).

**TABLE 1:** The distribution of clinical features in three cohorts.

Covariates	Type	Train cohort	Test cohort	Entire cohort
Age	≤65	43 (50%)	46 (53.49%)	89 (51.74%)
Age	>65	43 (50%)	40 (46.51%)	83 (48.26%)
Gender	FEMALE	40 (46.51%)	38 (44.19%)	78 (45.35%)
Gender	MALE	46 (53.49%)	48 (55.81%)	94 (54.65%)
Stage	Stage I	7 (8.14%)	13 (15.12%)	20 (11.63%)
Stage	Stage II	74 (86.05%)	68 (79.07%)	142 (82.56%)
Stage	Stage III	1 (1.16%)	2 (2.33%)	3 (1.74%)
Stage	Stage IV	2 (2.33%)	2 (2.33%)	4 (2.33%)
Stage	unknow	2 (2.33%)	1 (1.16%)	3 (1.74%)
T	T1	2 (2.33%)	4 (4.65%)	6 (3.49%)
T	T2	8 (9.3%)	15 (17.44%)	23 (13.37%)
T	T3	74 (86.05%)	64 (74.42%)	138 (80.23%)
T	T4	1 (1.16%)	2 (2.33%)	3 (1.74%)
T	unknow	1 (1.16%)	1 (1.16%)	2 (1.16%)
N	N0	26 (30.23%)	21 (24.42%)	47 (27.33%)
N	N1	57 (66.28%)	64 (74.42%)	121 (70.35%)
N	unknow	3 (3.49%)	1 (1.16%)	4 (2.33%)
M	M0	38 (44.19%)	38 (44.19%)	76 (44.19%)
M	M1	2 (2.33%)	2 (2.33%)	4 (2.33%)
M	unknow	46 (53.49%)	46 (53.49%)	92 (53.49%)

### 4.4 Functional enrichment

**FIGURE 7:**
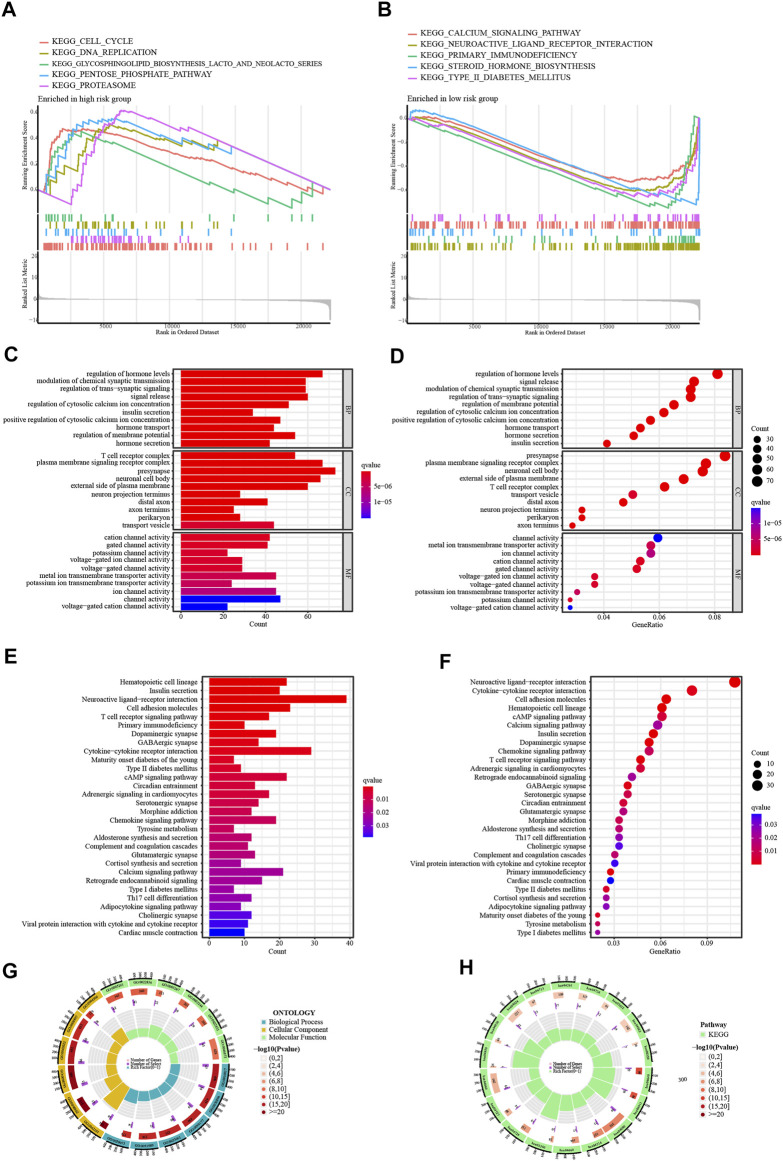
Enrichment Analysis of GSEA. **(A,B)** GSEA of the top 5 up-regulated and down-regulated KEGG signaling pathways enriched in high and low-risk groups. Enrichment Analysis of risk differentially expressed genes. **(C,D)** The bar and bubble plots of GO terms. **(E,F)** The bar and bubble charts of KEGG terms. **(G,H)** The GO and KEGG enrichment circle plots.

Initially, GSEA shows that high-risk patients are predominantly enriched in the signaling pathways of calcium signaling pathway, primary immunodeficiency, and type II diabetes mellitus (Figures 7A, B). Malignant activities such as cell proliferation are more active in high-risk patients. To further explore the functional enrichment of risk differentially expressed genes, a risk difference analysis was performed with the threshold set at logFC = 1, *p* < .05. Among them, the GO terms with the highest enrichment were, biological process: regulation of cytosolic calcium ion concentration; cellular component: T cell receptor complex; and molecular function: metal ion transmembrane transporter activity (Figures 7C, D). KEGG terms were mainly enriched in the T cell receptor signaling pathway and the cAMP signaling pathway (Figures 7E, F). The GO and KEGG enrichment circle plots show the number of differential genes enriched in the top eighteen items (Figures 7G, H).

### 4.5 Tumor mutation load analysis

**FIGURE 8:**
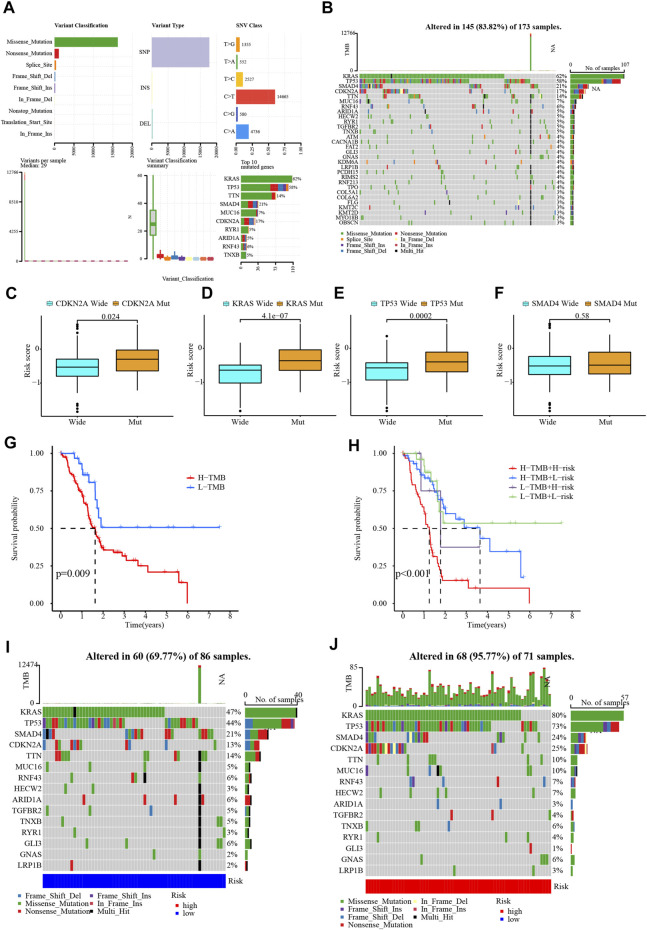
Mutation analysis of samples. **(A,B)** Waterfall plot for the PC patients in TCGA database. **(C–F)** The relationship between CDKN2A, KRAS, TP53, and SMAD4 gene mutation with risk score. **(G,H)** The relationship between TMB and PC survival. **(I,J)** Waterfall plots in different risk groups.

The most common variant classification among all mutations was missense mutation, and the most frequent variant type was SNP (Figure 8A). Mutations occurred in 145 of 173 PC samples (83.82%), with 62% of the samples harboring mutations in KRAS and 58% of the samples harboring mutations in TP53 (Figure 8B). PC patients carrying mutated KRAS, TP53, and CDKN2A had higher risk score than wild-type patients, while SMAD4 mutation was not associated with risk (Figures 8C–F). The TMB of the PC samples in the TCGA database was scored and they were categorized into two mutation groups. Mutations have a significant impact on survival time, with patients in the low-mutation group enjoying a much longer survival time than their counterparts (Figure 8G). Then, combining signature risk and TMB, we found that patients with high TMB from the high-risk group suffered the worst prognostic outcome (Figure 8H). The mutation landscape maps were dramatically diverse among patients with different risks, with a substantially higher mutation frequency in high-risk patients than in low-risk patients. The frequency of KRAS mutation was (80% vs. 47%) and TP53 mutation was (73% vs. 44%) in patients with high and low risks (Figures 8I, J).

### 4.6 Relationship between signature with immune infiltration

**FIGURE 9:**
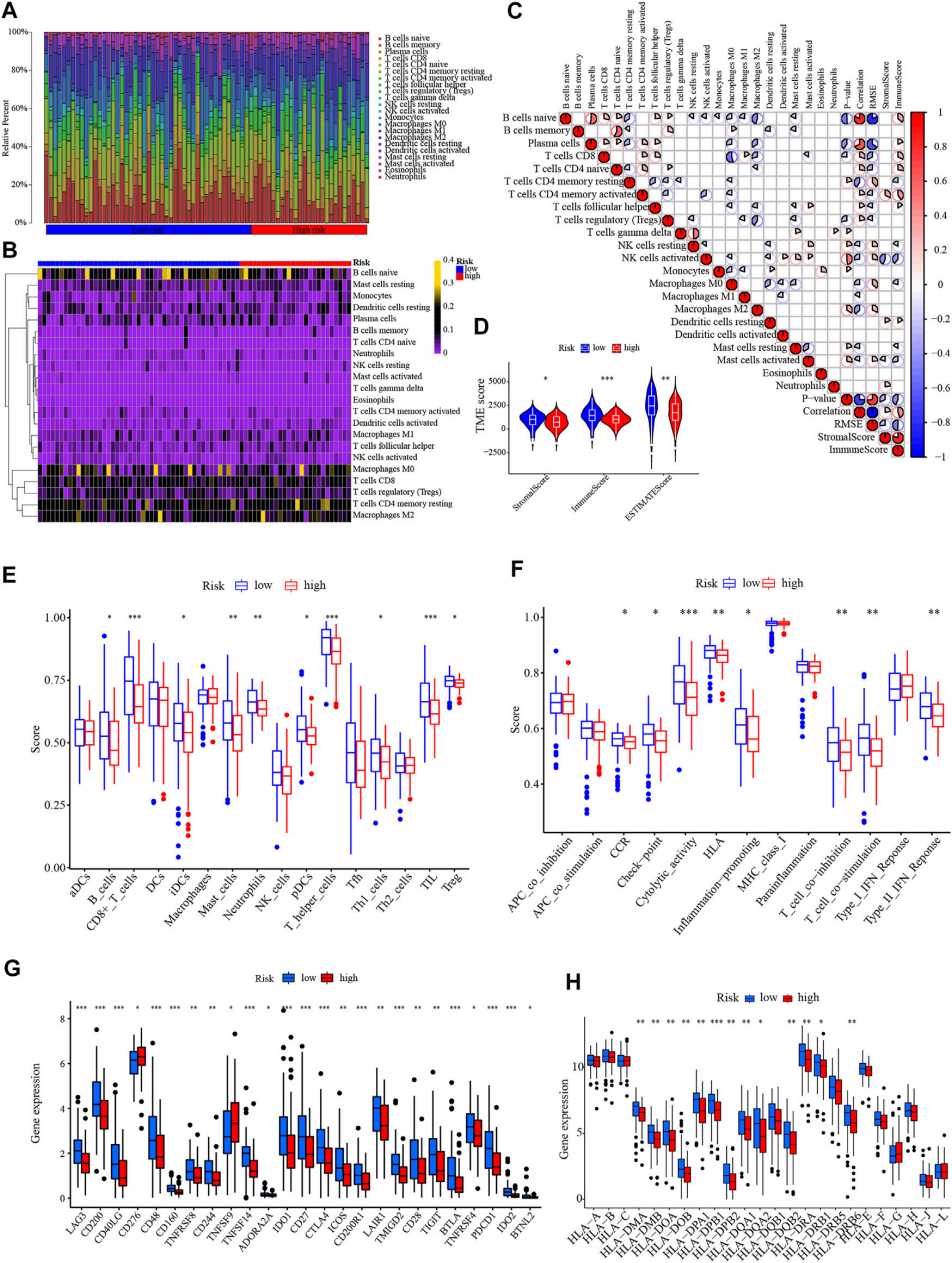
Immune infiltration analysis. **(A,B)** The bar chart and heatmap revealed the percentage of 22 infiltrating immune cells in the TCGA. **(C)** The correlation between infiltrating immune cells. **(D)** Comparison of the Stromal, Immune, and ESTIMATE scores between different risk groups. **(E,F)** The ssGSEA of the proportion of immune cells infiltration and immune function in different risk groups. **(G)** The differential expression of immune checkpoints between low- and high-risk groups. **(H)** The differential expression of HLA genes between different risk groups. *p < .05, **p < .01; ***p < .001; ns, not significant.

The proportion of immune cells varied significantly among PC samples in the TCGA (Figures 9A, B). Pearson’s correlation heatmap suggested that the correlation differed among immune cells. There was a significantly positive correlation between B cells memory and CD4^+^ T cells naive, while CD8^+^ T cells were markedly negative related to Macrophages M0 (Figure 9C). The stromal score, immune score, and ESTIMATE score were all at a much lower level in the high-risk sample (Figure 9D). The ssGSEA revealed that the infiltration levels of B cells, CD8^+^ T cells, iDCs, Mast cells, Neutrophils, pDCs, T helper cells, Th1 cells, TIL, and Treg were remarkably lower in high-risk patients (Figure 9E). In high-risk samples, immune functions of CCR, checkpoint, cytolytic activity, HLA, inflammation promoting, T cell coinhibition, T cell costimulation, and type II IFN reponse were less active in high-risk patients (Figure 9F). The ssGSEA results are consistent with the trend of lower a immune score. Given that checkpoint and HLA levels were lower in high-risk patients, checkpoint and HLA gene expression levels were then assessed. Overall, most of the immune checkpoints were substantially lower in the high-risk group, including CTLA4, PD-1 (also known as PDCD1, or CD279), and others (Figure 9G). Similarly, the vast majority of HLA genes were at low levels in high-risk patients (Figure 9H).

### 4.7 Drug susceptibility analysis

**FIGURE 10:**
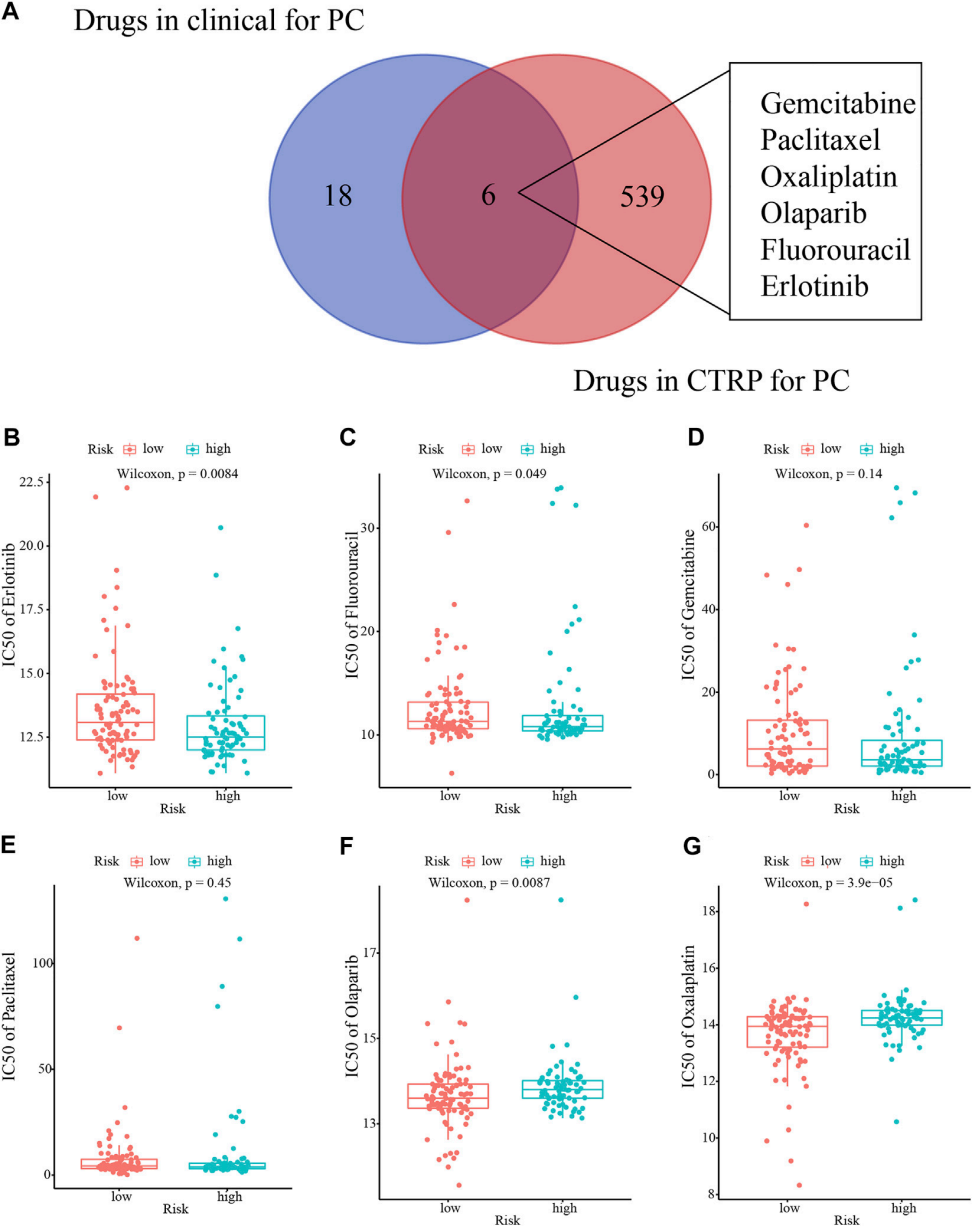
Drug sensitivity analysis. **(A)** Venn diagram of the clinical use of the drugs for PC. **(B–G)** The sensitivity to six drugs (Erlotinib, Fluorouracil, Gemcitabine, Paclitaxel, Olaparib, and Oxaliplatin) in different risk patients.

Initially, we identified six clinically used drugs (Gemcitabine, Paclitaxel, Oxaliplatin, Olaparib, Fluorouracil, and Erlotinib) from the candidate drugs for PC in the CTRP database (Figure 10A). High-risk patients were more sensitive to Erlotinib and Fluorouracil, while the sensitivity to Olaparib and Oxaliplatin was higher in low-risk patients (Figures 10B–G).

## 5 Discussion

PC is expected to be the second major cause of cancer mortality by the third decade of the 20th century, imposing a huge burden on social development and the physical and mental health of the population (Rahib et al., 2014; Siegel et al., 2021) (33433946, 24840647). The strong relationship between lncRNAs with cancer speaks for itself, and the potential of lncRNAs as accurate diagnostic, therapeutic, and prognostic biomarkers for malignancy is enormous (Ponting et al., 2009; Hauptman and Glavac, 2013) (23443164, 19239885). Consequently, we worked to establish a CRLncs prognostic signature for prognostic prediction of PC to guide personalized clinical management of patients.

In the current study, univariate Cox regression analysis identified 46 CRLncs associated with PC prognosis and developed a 6-CRLnc signature for PC. The model classified patients into two levels of high- and low-risk, with low-risk patients enjoying better clinical outcomes and this risk score being an independent prognostic factor for PC. In this study, six promising lncRNAs were identified, namely FAM83A-AS1, AL137186.1, SENCR, PAN3-AS1, Z97832.2, and AL139243.1. Recent studies reported that lncRNA FAM83A-AS1 exerted an important role in the proliferation, migration, invasion, autophagy, and progression of epithelial-mesenchymal transition (EMT) in lung adenocarcinoma (Chen et al., 2022; Huang et al., 2022; Zhao et al., 2022) (35002507, 35164653, 35635086). The biological roles of lncRNA SENCR are mainly in stabilizing vascular endothelial cell adhesion junctions and attenuating the proliferation and migration of vascular smooth muscle cells (Zou et al., 2015; Lyu et al., 2019; Song et al., 2022) (30584103, 35351345, 26349960). Ping et al. found that PAN3-AS1 was negatively associated with the prognosis of PC (Ping et al., 2022) (35330729). Nevertheless, no relevant reports on AL137186.1, Z97832.2, and AL139243.1 have been reported in cancer. The current study did not explore the potential functions and molecular mechanisms of these lncRNAs, but to a certain extent, it provided a theoretical basis for the link between lncRNAs with PC.

Replication immortality and cell cycle dysregulation are vital hallmarks of cancer cells, and DNA replication is a central process in cell proliferation (Hanahan and Weinberg, 2011; Qu et al., 2017) (21376230, 28182015). In the present study, GSEA results suggested that cell cycle and DNA replication related signaling pathways were more active in high-risk patients. Hence, high-risk patients exhibited more active tumor proliferative activity and faster tumor growth and progression, which is consistent with their higher malignancy. GSVA indicates that the Notch signaling pathway is more active in PC patients of the C2 subtype. The Notch signaling pathway has a vital role in cell development and contributes to cancer development and progression by fundamentally affecting cellular processes such as differentiation, proliferation, or migration (Reicher et al., 2018) (29409809). In Mullendore’s work, the Notch signaling pathway was identified to be involved in tumor initiation and maintenance of PC (Mullendore et al., 2009) (19258443). Downregulation of the Notch signaling pathway in PC facilitates inhibition of PC cell growth and invasion (Wang et al., 2006) (16510599). This implies that PC patients with the C2 subtype show a greater propensity for tumor progression and is consistent with a worse prognostic outcome.

Naturally, this study assessed whether the risk score was associated with PC gene mutation. The current study revealed that KRAS, CDKN2A, and TP53 mutations occurred at a considerably higher frequency in high-risk patients than in the corresponding groups. Among them, KRAS mutations and CDKN2A alterations were early events in the occurrence of PC (Kamisawa et al., 2016) (26830752). Mutations in the KRAS gene will permanently activate KRAS proteins that induce cell proliferation, migration, transformation, and survival by triggering various intracellular signaling pathways and transcription factors (Buscail et al., 2020) (32005945). The presence of KRAS mutation was related to an inferior prognosis for PC patients regardless of whether they underwent radical surgery (Buscail et al., 2020) (32005945). Whereas, mutation of CDKN2A increases the risk of PC (Goldstein et al., 1995) (7666916). In human cancers, TP53 is the most prevalently mutated gene, and wild-type TP53 is a pivotal tumor suppressor gene in cancer. Nevertheless, about half of human cancers harbor TP53 mutations (Harris and Hollstein, 1993; Muller and Vousden, 2014; Yue et al., 2017) (24651012, 8413413, 28390900). Numerous experiments have confirmed that mutated TP53 will acquire malignant biological functions in tumorigenesis, including promoting tumor cell survival, proliferation, migration, and invasion, enhancing chemoresistance, and promoting cancer metabolism (Dittmer et al., 1993; Brosh and Rotter, 2009; Muller and Vousden, 2013; Zhang et al., 2013) (23263379, 19693097, 8099841, 24343302). Thereby, high-risk PC patients are expected to develop disease progression.

A considerable amount of studies have covered that the infiltration of immune cells in the TME is intimately related to the prognosis of various cancers (e.g., ovarian, pancreatic, liver, breast, and colorectal cancers) (Zhang et al., 2003; Ino et al., 2013) (12529460, 23385730). B cells play an influential and complex role in the host’s immune response to malignancy but are often underappreciated. Given the reports, B cells can act both as antigen-presenting cells to activate T cell responses to tumor cells and also produce tumor-specific antibodies to bolster anti-tumor immunity (Fremd et al., 2013; Ko et al., 2020) (32730744, 24073382). Dendritic cells (DCs) are the most potent antigen-presenting cells in the body and play a pivotal role in the immune response, but are often suppressed in TME (Kranz et al., 2016; Chen et al., 2018) (27281205, 29290766). Interstitial dendritic cells (iDC) could bind antigens and stimulate T lymphocyte responses, posing a significant threat to organ graft survival after organ transplantation (Hart and McKenzie, 1990) (2152498). The impaired activity of plasmacytoid dendritic cells (pDC) is associated with immunodeficiency status or inefficient immune response to tumors (Reizis, 2019) (30650380). Regulatory T cells (Treg) exhibit a strong suppressive capacity and are critical mediators of tumor-associated immunosuppression (Liu et al., 2016; Plitas and Rudensky, 2016) (27590281, 26787424). Yet other studies pointed to the opposite function of Treg, that of tumor restriction. Studies suggested that Treg may promote tumor development by limiting anti-tumor immunity and also limit tumor development by restricting the mesenchymal environment required for its growth and metastasis (Tzankov et al., 2008; Liu et al., 2016) (26787424, 18223287). The exact role of Treg in the PC microenvironment is not yet fully understood, and the current study found higher levels of Treg infiltration in PC patients with low risk, which may be explained by the potential for Treg to limit PC cell growth and metastasis hence limiting tumor progression. The cytotoxic potential of CD8^+^ T cells is the backbone of current immunotherapy, and their high infiltration levels are associated with improved OS in cancer (Manzo et al., 2020; Orhan et al., 2020) (32491160, 32334338). High infiltration of tumor-infiltrating lymphocytes (TIL) could provide a rationale for differentiating immunogenic hot tumors from degenerative or cold tumors (Orhan et al., 2020) (32334338). The presence of TIL and high infiltration of CD8^+^ T cells often predicts response to immunotherapy and prognosis (Galon and Bruni, 2019; Orhan et al., 2020) (30610226, 32334338). The present study observed markedly higher levels of both CD8^+^ T cells and TIL in low-risk PC patients, with additional higher levels of expression of immune checkpoints like CTLA4 and PD-1, which seems to imply that patients with low-risk could benefit more from receiving immunotherapy. Of course, the currently accepted view is that high levels of CD8^+^ T cells and TIL infiltration do not represent an absolute enhancement of the efficacy of immunotherapy for PC and that the activity and depletion of immune cells, as well as the nature of TME, profoundly influence the response to immunotherapy (Galon and Bruni, 2020) (31940273). This could also explain, to some extent, the failure of patients with different risk PC to show significantly different responses to immunotherapy in the current study. Overall, the road to tangible benefits from immunotherapy for PC patients is difficult and urgently awaits further exploration.

Indeed, the current study has some limitations. The reliability and stability of this prognostic signature should be further validated in a prospective study at a large PC research center. All these concerns should be refined in future studies.

## 6 Conclusion

Novel lncRNA signature and nomogram for cuproptosis were constructed to predict the prognosis of PC patients. Molecular characterization based on this model provides a new perspective on the progression and subtype of PC. Differences in immune cell infiltration and immune checkpoint expression may be an important indication for the prognosis and treatment of PC patients. The differences in drug sensitivity might be a promising opportunity for the treatment of different risk PC patients.

## Data Availability

The datasets presented in this study can be found in online repositories. The names of the repository/repositories and accession number(s) can be found in the article/Supplementary Material.

## Nomenclature

PC: pancreatic cancer
LncRNAs: long non-coding RNAs
CRGs: cuproptosis-related genes
CRLncs: cuproptosis-related lncRNAs
TCGA: the Cancer Genome Atlas
OS: overall survival
ssGSEA: single-sample gene set enrichment analysis
LASSO: least absolute shrinkage and selection operator
ROC: receiver operating characteristic
AUC: area under the curve
RCD: regulated Cell Death
PFS: progression-free survival
DCA: decision curve analysis
GSEA: gene set enrichment analysis
GO: gene Ontology
KEGG: kyoto Encyclopedia of Genes and Genomes
TMB: tumor mutation burden
GSVA: gene set variation analysis
FDR: false discovery rate
PCA: principal component analysis
t-SNE: t-distributed stochastic neighbor embedding
HR: hazard ratio
CI: confidence interval.
